# A Case of Purpurea Hemorrhagica

**Published:** 1848

**Authors:** W. Wadsworth

**Affiliations:** Racine, Wisconsin


ARTICLE IV.

*A Case of Purpurea Hemorrhagia.* By W. Wadsworth, M.D., of Racine, Wisconsin.

I was called, on the 28th of November, 1847, to see a little girl of Dr. J. Shepard, aged eight years, of a delicate nervous constitution. She had suffered, a few months since, an attack of remittent fever, from which she had fully recovered and enjoyed pretty good health. Three days before, she was taken with pain and swelling of the right ancle, extending up the gastrocnemius muscle, with slight febrile disturbance, for which a dose of calomel and rhei was given, and a blister applied to the ancle. The next day the other extremity was attacked in like manner, with chills, sickness of stomach and vomiting, and a depressed state of the general system. There was now, also, observed little spots of a vivid red color, scattered about, upon both legs; the right wrist had, during the night, become swelled and painful. Quinine, Dover, and hyd. cum creta, had been given every few hours. At this time the pulse was frequent and feeble, the breath fetid, murcurial odor evident, the spots on the skin enlarging, some had attained the size of a shilling, others that of a dollar, of a bright red color, which could not be affected in the least, by pressure. It appeared to be a case of acute Rheumatism complicated by Purpurea Hemorrhagia. Continued the Dover with decoction of bark and sulphuric acid, every three hours.

*November 29th.*—The rheumatic affection had materially improved, swelling and tenderness nearly gone. There was slight fever, pulse frequent and feeble. The bowels had moved some five or six times, with occasional vomiting during the night. Laudanum, with a few grains of acetate lead, had been given. There was much complaint of the mouth, the cheeks becoming swollen. In the after part of the day, blood was discharged from the bowels, amounting to several spoonfuls In consultation, it was determined to make trial of the oil of turpentine—33, with as much castor oil, was given. It was immediately rejected. A little time after, a desert spoonful of the turpentine, in peppermint water, was given and retained, and four hours after, a smaller dose repeated.

*November 30th.*—The bowels have moved several times during the night, nearly half pint of blood in the discharges, great prostration mark the general symptoms. The purpurea on the extremities declining, large patches nearly gone, leaving a yellowish brown shade where they existed. The turpentine was continued in smaller doses, with bark and wine, and sulphuric acid.

*December 1st.*—The bowels loose, but a little blood in the discharges, the mercurial action on the mouth very severe, the cheeks greatly swollen, the sputa streaked with blood, astringent gurgles freely used, and Dover and camphor, bark and wine continued.

*December 2d.*—Hemorrhage increasing from the mouth, with extreme fetor of the breath. The chloride of soda used as a gurgle, and some ulcerated points in the mouth cauterised with the nitrate of silver, during the night. Gave the acetate of lead and opium, every four hours—the general treatment the same.

*December 3d.*—The hemorrhage still continuing from the mouth, the turpentine was again resumed in free and purgative doses, with marked effect over this troublesome symptom; the strength endeavored to be sustained by strong beef tea, wine, quinine, and bark.

*December 4th.*—Purpurea spots have showed themselves upon the eye-lids, arms, and ears, some of a red, others of a purple hue, no hemorrhage from the mouth or bowels, no fever, nor has there been for several days. A dose of turpentine and castor oil, to be given during the night—the nitrate of silver, one-half grain, every four hours, and the general treatment the same.

*December 12th.*—Since our last report, the patient has been steadily improving. The purpurea spots, except a few about the elbow, have disappeared; no hemorrhage has been present. The nitrate of silver was continued but a short time. The oil of turpentine occasionally given, and a steady perseverance in the bark and wine. Her convalescence is complete. In reviewing this interesting case, it is a matter of regret, that mercury, in the early stage and before purpurea was fully established, had been given. The severe constitutional irritation consequent upon it, as well as the hemorrhage from the mouth, which followed, was calculated greatly to embarrass, and caused for a time a fearful gloom to rest upon the prognosis. The turpentine had *a marked and beneficial effect upon the disease*, as seen in the rapid disappearance of the spots on the skin and afterwards, the hemorrhage from the mouth, which for more than twelve hours, resisted large doses of the acetate of lead, was speedily controlled by it. I have seen but little of this disease, two cases only before, both of which were fatal. In neither, was the turpentine used. Dr. Neligan's * report of four cases successfully treated by it, induced me to give it a trial, and the result has been satisfactory. In what manner it operates, whether specifically on the blood itself or in giving tone to the solids, and especially the capillary vessels, I am unable at present to determine. But that it will be found a remedy of great value in this dangerous and often fatal disease, I cannot entertain a doubt.

* Braitwaite's Retrospect, No. 12, p. 133.

